# Automated high-throughput Raman detection of fluidic samples: measurement setup and methods preventing air bubble interference

**DOI:** 10.1007/s00216-026-06612-0

**Published:** 2026-06-12

**Authors:** Jingyi Chen, Jiarui Wang, Elmar Schuck, Gang Wang, Joey Studts, Matthias Franzreb

**Affiliations:** 1 Boehringer Ingelheim Pharma GmbH/Co. KG, Biberach an Der Riss, Germany; 2 Institute of Functional Interfaces, Karlsruhe Institute of Technology, 76344 Eggenstein-Leopoldshafen, Germany

**Keywords:** Liquid handling station, Raman spectroscopy, Flow-cell injection, Automation, Air-free detection, Bubble interference

## Abstract

Raman spectroscopy has widely been recognized as an analytical tool of high potential in biopharmaceutical downstream processing, and the use of flow cells has been suggested for use in in situ or in-line Raman spectroscopy measurements at least for development-scale processes. However, there remains a need for Raman analysis of samples that cannot be accessed through an in-line monitoring system, particularly for low-volume samples in micro-to-milliliter range. This article presents a new setup that enables automated Raman measurements of liquid samples with low injection volume of 300 µL. A designed adapter links a Tornado Micro Flow Cell to a Tecan liquid handling system, allowing direct sample delivery. This work focuses on a set of methods implemented on this setup to address the issue of air bubbles, which can significantly interfere with Raman signal quality during measurement. These methods include adapted residence time, a three-step purging/cleaning method, combined with a vertical and upflow flow-cell orientation. A series of experiments demonstrates the effectiveness of these methods in reducing air bubbles and improving spectral robustness. The presented setup and methods enable automated high-throughput Raman detection of fluidic samples with small injection volumes and high-quality spectral data.

**Graphical abstract:**

**Figure:**
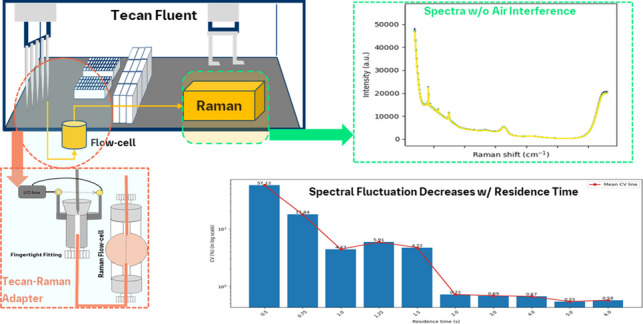

## Introduction

Raman spectroscopy has been pointed out as a promising tool for Process Analytical Technology (PAT) in real-time monitoring of downstream process (DSP) development [1]. Over the past decade, advancements in measurement setups have enabled continuous, non-invasive Raman measurements without the need for sampling in DSP. To quantify components of interest, model calibration is an essential process that establishes a chemometric relationship between analytical references and spectroscopic responses. In general, Raman data acquisition for calibration in biopharmaceutical process development can be performed by two methods. The first involves in-line measurements using a flow cell [2, 3] or on-/in-line measurements with an immersion probe [4–6]. This method requires multiple reference analytics for each sample, depending on the target quality attributes to be calibrated. In addition, the achievable calibration range and dataset size are limited by the number of samples that can be collected during processing. The second method relies on off-line sample measurement. It involves preparing samples by mixing feedstock solutions with known component compositions and subsequently loading them into a flow cell, a cuvette [7], or onto a carrier [1]. Calibrating multivariate chemometric models is commonly achieved through this off-line measurement of pre-designed samples with known component concentrations [7–13]. Sample numbers may range from 50 to several hundred depending on several factors: the number of influencing factors in the studied process, the selected model algorithm, and the desired calibration range for each factor. Preparation and measurement of large numbers of samples often result in bottlenecks. These bottlenecks can be overcome by developing an automated high-throughput setup [1] for Raman data acquisition.

We recently reported the integration of Raman spectroscopy with a Tecan liquid handling station using an in-house-made connector [13–15]. This connector enables sample injection from the Tecan needle directly into the flow cell without leakage, which facilitates automated Raman measurements on the liquid handling station. However, the connector requires manual fabrication, which is impractical for routine use and results in limited reproducibility. Additionally, air bubbles frequently form during the injection process, leading to spectral fluctuations and affecting the quality of Raman data. The primary challenge in this setup is to establish a bubble-free fluidic connection between the flow cell and the automation station without leakage.

We enhanced the connector by introducing a custom-designed, 3D-printed adapter between Tecan needle and flow cell. Building on this adapter, we developed three methods to prevent air from entering the Raman flow cell and/or to expel any bubbles that may have formed. These strategies include (1) optimizing residence time, (2) arranging the flow cell vertically to enable bottom-to-top flow, and (3) implementing a three-step purging/cleaning procedure. We then assessed the effectiveness of the three-step purging and cleaning method, with the flow cell in both vertical and horizontal orientations.

## Materials and methods

### Integrated Raman-Tecan system

**Fig. 1:**
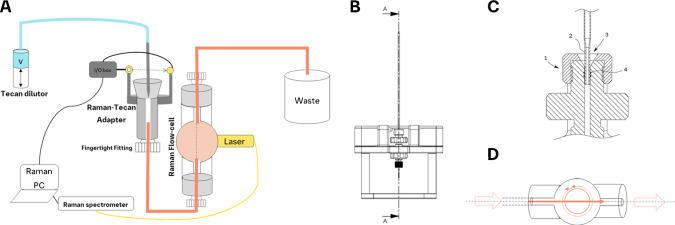
**A** Schematic representation of the connected Raman-Tecan system through a 3D-printed adapter. **B** CAD drawing of the adapter including the Tecan dispensing needle. **C** Cross-sectional view of the Raman-Tecan adapter assembly. This view shows: 1, adapter body; 2, injection needle; 3, receiving port; 4, seal element. **D** Two fluid profiles in a Raman flow cell during sample injection

Figure 1A illustrates the integrated system linking a HyperFlux Pro Plus Raman spectrometer to a Tecan Fluent 780 liquid handling station. The Tecan system features a Flexible Channel Arm (FCA) with eight fixed tips (Tecan, Männedorf, Switzerland). The Raman system includes a 785-nm emission laser, Hudson Flow Probe, and a 45-µL micro flow cell (Tornado Spectral Systems, Ontario, Canada). Raman spectra are continuously acquired every 7.5 s (500 ms exposure, 15 averages) across 200–3300 cm^−1^, with a laser power of 495 mW. As shown in Fig. 1B, one Tecan tip is inserted in a custom 3D-printed adapter through a lip. The bottom threaded hole is designed to fit tightly with a PEEK tubing (L = 20 cm, ID = 0.5 mm). Figure 1C presents a cross-section of the adapter. This adapter ensures a replaceable sealed O-ring (part 4 in Fig. 1C) connecting the Tecan dispensing needle (part 2) and the tubing. The top lip (part 1) is screwable for the purpose of replacing the O-ring. The samples flow vertically through an upflow-configured Raman flow cell and are directed to the waste container. Figure 1D illustrates two fluids in a horizontally placed flow cell during sample injection: a straight fluid from inlet to outlet and a circular fluid along the cylindrical wall of the flow cell.

For Raman detection, one Tecan tip aspirates the sample (from 300 to 1000 µL). Then, the tip afterwards dispenses the sample above the adapter at a controlled velocity. A light sensor-based needle detector [13–15], mechanically coupled to the adapter, uses a light barrier to track needle insertion and retraction. The recorded timestamps are used to identify and extract relevant Raman spectra from continuous acquisition.

### Residence time at the dispensing tip

During sample injection, the dispensing tip is moved in the adapter and positioned directly above the inlet of a tubing part connected to the flow cell. Although an O-ring provides a sealed fluidic connection, the two components (the dispensing tip and the tubing) are not physically joined. We discovered that the linear flow velocity through this fluidic connection has an effect on the formation of bubbles: higher linear flow velocities increase the probability of introducing air bubbles at the connection point. To quantify this effect, we introduced the term ԏ “residence time” to describe the time required to pump the volume of a single fluidic sample located at the dispensing tip.

To investigate the effect and determine the optimal residence time, we varied the residence time across ten distinct values: 0.50, 0.75, 1.0, 1.25, 1.50, 2.0, 3.0, 4.0, 5.0, and 6.0 s. Each residence time was measured with 30 replicates. On the proposed Tecan-Raman setup, a total of 300 sample measurements were performed with the varied residence times in a fully randomized sequence. The flow cell was initially filled with purified water without introducing any air bubbles. The dead volume from the tubing inlet to the flow cell is approx. 84 µL (39 µL in the tubing and 45 µL in the flow cell). An injection volume of 100 µL of purified water was selected to ensure that any air bubble formed during the injection process would become trapped inside the flow cell and therefore detected by the Raman spectrometer. After measuring each sample, a three-step purging and cleaning method was carried out to flush any air bubbles out from the flow cell. This method is described in detail in.” For both the injection and flush phases, Raman acquisition was conducted for one minute, yielding an averaged spectrum for each phase.

To evaluate the performance at different residence times, the coefficient of variation (CV) was used to measure the dispersion of the water spectra at all Raman shifts for measurements with the same residence time. The CV is calculated as the ratio of the standard deviation to the mean, expressed as a percentage.

### Three-step flush method with vertical upflow

To facilitate automated sample detection, the previous sample component must be completely removed to prevent interference from residual components or air bubbles during subsequent sample measurement. For this reason, we developed a three-step purging and cleaning method designed to flush out the previous sample and expel any air bubbles from the flow cell. This method includes two purge steps and one wash step, each using 1 mL of purified water. The first purge applies a residence time of 4 s to remove large bubbles, followed by a residence time of 2 s to clear smaller ones. Finally, a slow wash with a residence time of 10 s keeps the cell clean and ready for the next sample.

Despite using the three-step method, small air bubbles were observed adhering to the rounded sides of the flow cell during the rapid purge. These lightweight bubbles became trapped in the flow cell due to the circular flow of fluid along the cylindrical wall of the flow cell, as shown in Fig. 1D. An efficient solution is to guide them along the straight flow path from inlet to outlet, preventing them from entering the circular motion where they tend to get trapped. To address this, we placed the flow cell in a vertical upflow orientation. This setup allows bubbles to rise towards the outlet and to be cleared more effectively during the purge step.

When the flow cell is placed vertically for upflow, the three-step flush method effectively cleans the flow cell and minimizes bubble interference during Raman measurements. To evaluate the long-term performance of this flushing method, we designed an experiment consisting of 100 replicates. In this experiment, we first injected air (standard injection volume of 300 µL) instead of water into the flow cell, and then applied the three-step flush method to verify its ability to independently remove the introduced air. For comparison, both the flush experiment and residence time experiment (“Residence time at the dispensing tip”) were repeated twice, once with a vertical upflow direction and once with a horizontal flow direction. Raman spectra were acquired during each injection phase (air measurement) and each flush phase (water measurement), with each phase lasting one minute to obtain the average spectrum. The CV value was also used as a metric to evaluate the effectiveness and performance of the method.

## Results and discussion

### Effective air removal via the three-step method

**Fig. 2:**
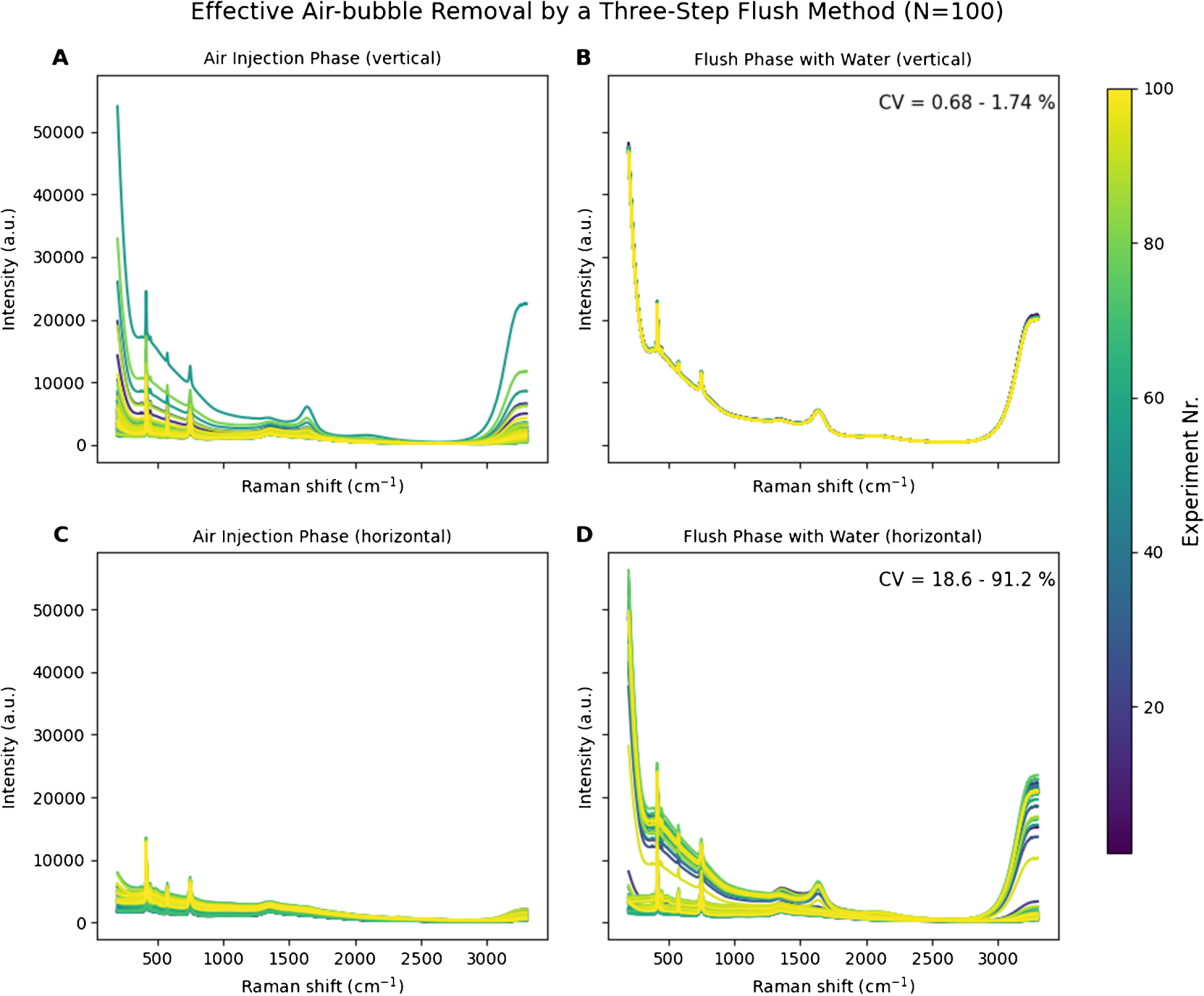
Performance comparison of the three-step flush method for removing air bubbles under vertical upflow and horizontal flow orientations (*N* = 100). Overlapping Raman spectra (*N* = 100) collected during the air-injection phase under vertical upflow (**A**) and horizontal flow (**C**). Overlapping Raman spectra (*N* = 100) collected during the water-flush phase under vertical upflow (**B**) and horizontal flow (**D**). The color gradient indicates the experimental sequence from 1 to 100. The coefficient of variation (CV) of the spectra collected during the flush phases is calculated at each Raman shift to quantify spectral fluctuations across the 100 experiments, assessing the effectiveness of the flush method under each flow orientation

As illustrated in Fig. 2, the Raman spectra were collected during the flush experiments (“Three-step flush method with vertical upflow”). In order to verify the effectiveness of the three-step flush method, air was initially introduced into the flow cell, as shown in Fig. 2A (vertical upflow) and Fig. 2C (horizontal flow). Obviously, the spectra in both sub-figures have varying degrees of air interference, as reflected by the decreased spectral baseline. This decrease is most noticeable across Raman shifts from 200 to 1800 cm^−1^ and from 3000 to 3300 cm^−1^. For instance, the set-up-specific sapphire peak at 418 cm^−1^ has an intensity of ca. 24,000 counts in the water spectra (Fig. 2B). However, its intensity decreases to around 12,000 counts during the air-injection phase (Fig. 2A and C).

After each air-injection, the three-step flush method was employed to remove the previously introduced air and to obtain a clean water spectrum. The corresponding water spectra are overlapped and shown in Fig. 2B (vertical upflow) and Fig. 2D (horizontal flow). In contrast to the air-injection phase, no significant decrease in the spectral baseline is observed during flushing under the vertical upflow (Fig. 2B). However, under horizontal flow (Fig. 2D), the spectra demonstrate significant and random fluctuations, which are not accumulated over time. Furthermore, in several experiments conducted under horizontal flow, only air spectra were collected, indicating a complete failure of the method to remove the air. Over 100 cycles of switching from air injection to water flushing, the post-flush water spectra obtained under vertical upflow show consistent behavior, achieving minimal fluctuation and a low coefficient of variation (CV) ranging from 0.68 to 1.74%. This finding demonstrates the high reliability and reproducibility of the flush method. In contrast, the same test performed under horizontal flow presents substantially larger variability, with CV value ranging from 18.6 to 91.2%.

These results demonstrate that the three-step flush method effectively removes air bubbles under vertical upflow and shows good long-term robustness. This method includes two purge steps (10 s and 4 s) followed by a wash step (~ 17–18 s). Including needle switching, the complete flushing process takes around 50 s and is both simple to implement and highly effective.

### Effect of residence time at dispensing tip

**Fig. 3:**
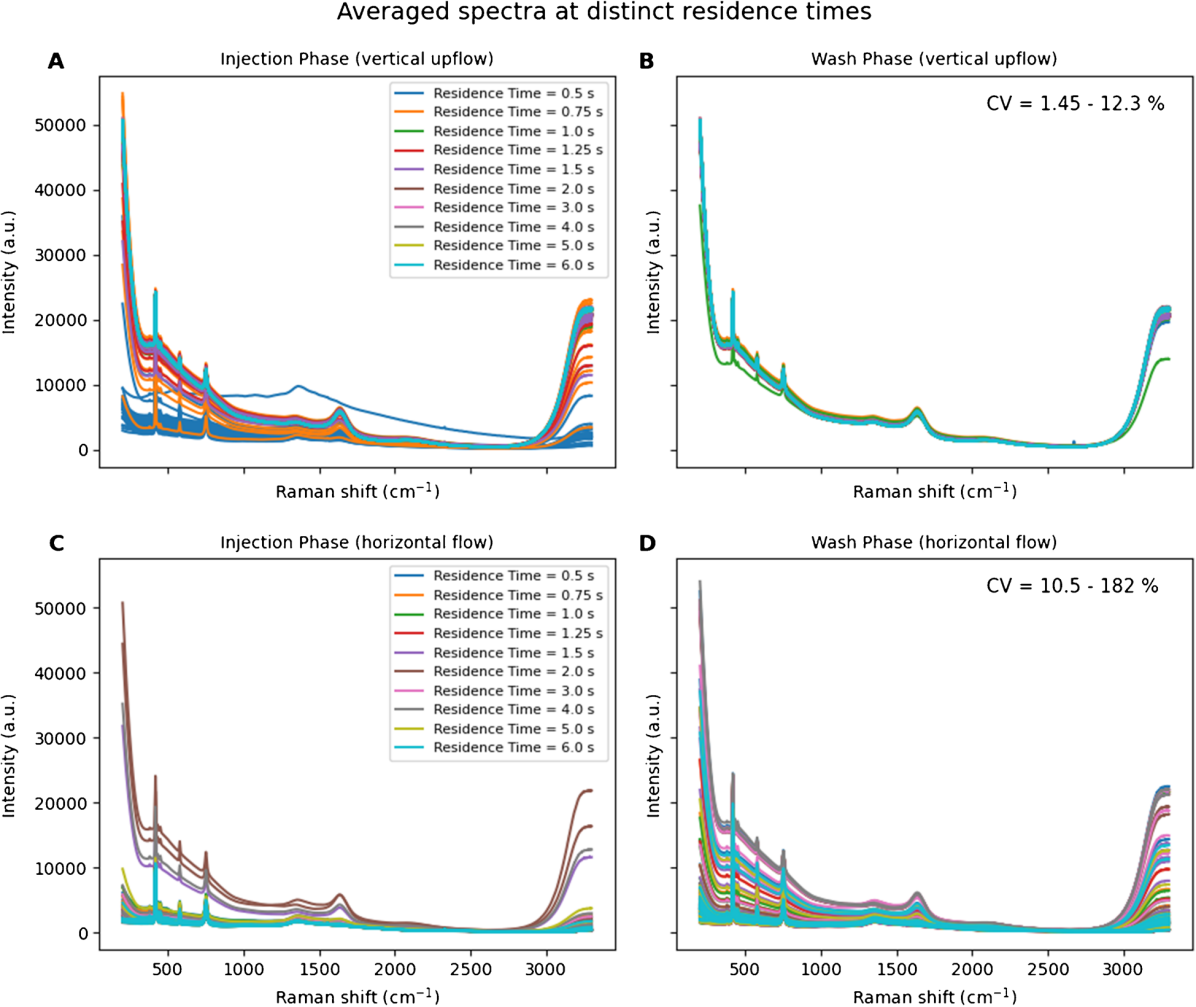
Bubble-interference comparison during water injection at ten distinct residence times (*N* = 300). Overlapping Raman spectra collected during water injection phases with vertical upflow (**A**) and horizontal flow (**C**). Effectiveness and performance of the flush method under vertical upflow (**B**) and horizontal flow (**D**). The legend represents the ten distinct residence times, each represented by 30 replicates. The coefficient of variation (CV) of the spectra collected during the flush phases is calculated at each Raman shift to quantify spectral fluctuations across the 300 experiments, assessing the effectiveness of the flush method under each flow orientation

To investigate the effect of residence time at the dispensing tip on air bubble introduction in the flowing system, we conducted a study consisting of 300 randomized injection-and-flush experiments using water. Instead of the standard 300 µL injection volume, we intentionally reduced the volume to 100 µL to detect the introduced air, as described in “Residence time at the dispensing tip.” Ten distinct residence times were evaluated, with 30 replicates per residence time. For all experiments, spectra were acquired during the injection and flush phases and are plotted in Fig. 3.

**Fig. 4:**
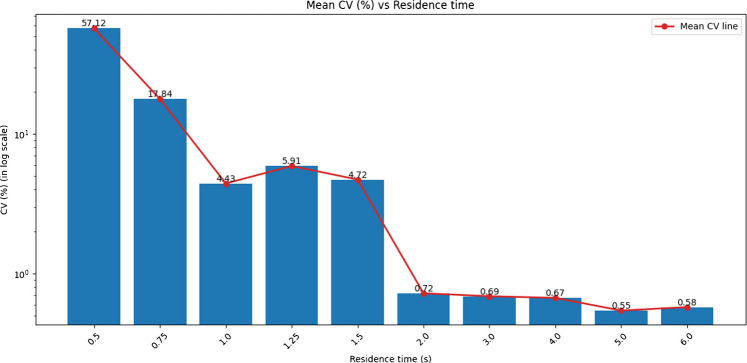
Averaged coefficient of variation (CV) with increasing residence time. The averaged CV is calculated from 30 replicates at each of the ten residence times and presented in a logarithmic scale

As shown in Fig. 3A, the baselines of the dark blue spectra decrease sharply and reach the lowest level, with one spectrum showing an abnormal offset. These observations indicate unstable water injection and unsatisfied quality of Raman measurements. When the residence time is increased to 0.75 s (orange lines), the spectral baselines continue to vary across experiments, suggesting different degrees of air interference. In contrast, with a residence time of 6.0 s, water spectra with less fluctuations are obtained, as indicated by the light blue lines. Because the spectra in the figures largely overlap, we grouped the spectra with the same residence time and calculated their CV values at each Raman shift to quantify the spectral fluctuations for comparison. Table 1 summarizes these CV values for all residence times during water-injection phases, including the average, minimum, maximum, and delta values. The average CV values are plotted with the residence time in the bar chart (Fig. 4). It is evident that the average CV value decreases with increasing residence time, with a particularly sharp reduction at residence times below 1.0 s. Between 1.0 and 2.0 s, the CV values reach a first plateau in the range of 4–6%. When the residence time is further increased beyond 2.0 s, the CV values decrease to a sec, lower magnitude below 0.8%. This value is comparable to the CV value range of 0.68–1.74% obtained using the successful flush method (Fig. 2B). The results demonstrate that a residence time above 2.0 s is the optimal range for injecting samples without introducing any air bubbles. On the automated setup, a residence time of 3.0 s is taken as a standard parameter.

**Table 1:** Overview of the averaged, minimum, maximum, and delta values of the coefficient of variation (CV) for each residence time during the injection phase. The CV is a dimensionless, relative measure of dispersion, calculated from 30 replicates at each condition

Residence time (s)	Mean CV (%)	Min CV (%)	Max CV (%)	Delta CV (%)
0.50	57.123	14.753	92.648	77.895
0.75	17.842	13.169	21.487	8.318
1.00	4.431	1.990	7.854	5.863
1.25	5.909	4.408	8.266	3.857
1.50	4.724	3.066	8.220	5.154
2.00	0.725	0.315	1.562	1.247
3.00	0.690	0.330	1.224	0.894
4.00	0.671	0.415	2.243	1.827
5.00	0.545	0.343	1.021	0.678
6.00	0.577	0.318	1.625	1.308

In the residence time experiments, we employed not only a fully randomized sequence but also the flush method to minimize confounding factors, such as air accumulation over time and over samples. Figure 3B shows clear water spectra collected after flushing, with CV value in a range of 1.45–12.3%. One spectrum (in green, ԏ = 1.0 s) obtains a slight drop in the spectral baseline. This indicates the presence of minor air interference, resulting in a higher CV value than in Fig. 2B. However, the trapped air was subsequently removed by the method itself and is not observed in the remaining spectra. These results demonstrate robust flush performance and confirm that no cumulative air-related effects occurred during the residence time experiments.

As in the flush experiments, the residence time experiments were repeated to verify method performance under horizontal flow. In contrast to Fig. 3A and B (vertical upflow), a strong air influence is observed during both injection and flush phases at all residence times in the horizontal configuration (Fig. 3C and D). In Fig. 3C, although several spectra (in dark red, grey, and violet) show only minor air-related fluctuations, the baseline of the majority of spectra is reduced to the lowest magnitude, which indicates measurements of full air. Moreover, these baseline reductions could not be removed by the flush method, as the spectra in flush phase show random and varied fluctuations (Fig. 3D) with CV values reaching up to 182%. These results clearly demonstrate that the horizontal configuration lacks long-term robustness and is unsuitable for automated detection.

### Setup improvements for automated Raman detection

In the automation setup reported by Wang et al. [13], the procedure of creating an interface is complex and non-standardized, often requiring multiple attempts. As an in-house hand-made consumable component, the interface of Wang et al. is manually drilled, resulting in limited reproducibility, robustness, and liability to wear. The fabrication process is technically demanding, and successful measurements are achieved only in a small fraction of attempts. After hundreds of injections, the interface might need to be replaced. In one of our attempts, a plastic fragment detached from the interface body and entered the flow cell, causing a critical failure during an overnight run. This contamination disrupted the sample measurements and continued until the flow cell was manually disassembled and cleaned. Compared to this former setup, the experiments in the present study comprised a total of 2000 needle injections (each experiment consisted of one sample injection and three flush injections) without need to replace any consumable element. The adapter was designed with CAD drawing and can be reproduced by 3D printing, enabling standardized production if required. The standardized production of the adapter, coupled with the long-term robustness of the measurement methods, enables reliable Raman measurements on the Tecan system. The duration of automated detection was reduced from 43 h with 169 samples [14] to just 6 h with 100 samples (a sample measurement of 2 min), significantly improving the efficiency of the high-throughput Raman detection.

This setup supports Raman measurements of samples stored in 2-mL tubes, 15- or 50-mL centrifuge tubes, and 96-well plates. It is suitable for a broad range of applications, including in-process control from process intermediates, freeze-thaw studies, stability studies, and the measurement of calibration samples for model development. For model calibration, an automated Raman calibration study [16] was previously performed on an ÄKTA system, where a quaternary valve and gradient were used for in-line sample mixing at a flow rate of 10 L/min with a flush volume of 15 mL per sample. In addition, the in-line Raman measurement required 1 mL, resulting in a total sample volume of 16 mL per sample. In contrast, the setup presented here reduces the required sample volume to as little as 300 µL per sample because of off-line mixture and lower dead volume, corresponding to a 98% reduction in material use and cost. However, for highly concentrated proteins, such as those from ultrafiltration, diafiltration, or formulation processes, even 300 µL may still represent a substantial material demand. The main reason is dispersion within the fluidic system, which requires a larger injection volume to avoid strong dilution by residual fluid in the flow cell. By comparison, Lange et al. [17] reported a cuvette-based automation system for upstream processes that enabled Raman measurements with only 50 µL per sample.

## Conclusion

This work presents an automated setup that links Raman flow cell with a liquid handling station to enable air-free sample injection and robust Raman measurement. Using a 3D-printed adapter, the setup provides a reliable fluidic connection and disconnection between the injection needle and the flow cell. Nevertheless, air bubbles can still be introduced and trapped in the flow cell. To address this, we developed a three-step purging/cleaning method as a standard procedure before/after each sample injection, in order to remove potential air bubbles and thereby minimize air interference in Raman measurements. Experimental results highlight the effectiveness and robustness of this method, showing that intentionally introduced air bubbles can be easily removed within 50 s. In addition, we showed that short residence times, especially less than 1 s, at the dispensing tip of the injection needle can introduce air bubbles. We further determined that a residence time greater than 2 s reliably prevents air bubble formation. Our results also indicate that both methods perform successfully only under one specific condition: a vertical upflow orientation of the flow cell. Overall, this study demonstrates that optimized residence time, flushing method, and flow-cell orientation are essential for ensuring bubble-free automated Raman measurements on liquid handing station and achieving high spectral quality and accuracy. These advancements mark a significant step toward implementing Raman spectroscopy as a PAT tool in downstream process development and manufacturing.

## Data Availability

The datasets generated and analyzed during the current study are not publicly available due to confidentiality and proprietary restrictions of the company and are therefore not available from the authors.
